# Medicinal Properties of Bromine and Its Compounds

**Published:** 1842-11-05

**Authors:** 


***Medicinal Properties of Bromine and its Compounds.***—Bromine was first used as a medicinal agent by M. Pourché, a townsman of Balard. Previous to this, a person named Desorgues, calling himself ***simple magistrat***, had written to the Academy of Medicine, proposing the employment of bromide of mercury in the treatment of syphilis.

M. Bonnet in a paper published in the ***Bulletin Géneral de Therapeutique***, July 1837, gives a complete digest of what had been then observed in France with regard to the medicinal uses of bromine and its compounds. He refers to the researches of M. Pourché. In a case of scrofulous enlargement of the glands of the neck in a woman of 22 years of age, who had been affected for seven years, a cure was effected in three months by the external and internal use of bromine. At first six drops dissolved in three ounces of water were given in the day, in three doses. Next day, ten drops were given. In ten days, the dose was increased to fourteen drops daily, and at last to thirty drops in the same quantity of water. Cataplasms, moistened with solution of bromine, were applied to the swellings. The same physician had great success in the treatment of scrofula by the internal and external use of the hydrobromate of potass.

M. Bonnet attributes great advantages to the bromide and sub-bromide of mercury, as constitutional remedies in comparison with corrosive sublimate and calomel. He states, that the sub-bromide has less action than calomel on the salivary glands, and more effect on the urinary secretion; that the former substance is therefore preferable where the idiosyncrasy of the patients renders them liable to salivation. After stating that the bromide is less soluble in water than the chloride, he recommends a solution in ether for medicinal use, and gives the observations of an Austrian physician, which prove the antisyphilitic virtues of the remedy, and contends that it is not so liable as corrosive sublimate to affect the head, chest, and stomach during its medicinal use.

In the cases of M. Fournet, already referred to, the therapeutic action of the bromine is exceedingly obscure. Frictions of bromine to the swollen articulations appear to have been useful, but as their application was conjoined with alkaline baths, and, moreover, the bromine was mixed with alcohol, which would soon change it into bromal and hydrobromic ether, these cases prove very little.

Magendie employs bromine and its preparations in scrofula, amenorrhœa, and in hypertrophy of the ventricles. He expresses his conviction that future observation will establish the great therapeutic powers of bromine.

Dr. Williams has used the bromide of potassium with success in cases of enlarged spleen. The first case which he gives is that of a boy, aged 14, admitted into St. Thomas' Hospital on the 13th of September 1833. Both the liver and spleen were enormously enlarged. Their edge was hard, and substance unyielding. The abdomen contained much fluid; the countenance was sallow and emaciated; the legs dropsical; belly protuberant. The prognosis was most unfavourable. After unsuccessful trial of the supertartrate of potass and iodide of potassium, the iodide of mercury was used. This last remedy removed the dropsy, but the liver and spleen remained enlarged. On the 13th of May, the patient commenced with a grain thrice a day of the bromide of potassium, which dose was gradually increased to four grains. On the 10th of July he became slightly jaundiced. From apprehension that the bromide might have caused this, he had doses of the sulphate of magnesia instead until the jaundice had disappeared, when, on the 11th of August, the bromide was recommenced in four grain doses. On the 15th the dose was increased to five grains thrice a day, and continued for fourteen months. Under this treatment, he gradually improved, and was dismissed with the liver and spleen only one-third of their former size.

In a second case of enlarged spleen with ascites, in a woman aged 30, the bromide could not be given in larger doses than four grains three times a day, on account of its tendency to disorder the bowels. After nine months treatment by the bromide, she was dismissed with the spleen still above the natural size. Dr. Williams gives two more cases in which the results were of the same character.

I shall give a summary of my own results.

*Case* 1.—Eczema of the legs and arms in a married woman aged 40, of strumous habits, the mother of a large family. The disease, an inveterate form of eczema, had lasted a twelvemonth; for six months she had been under the care of Mr. Brady of Gateshead, who had tried a variety of remedies without success. The eruption became moist every morning about three o'clock, and continued so for an hour, it then turned dry, and was accompanied by heat, redness, and itching. A saturated solution of bromine mixed with water until it ceased to give pain, and applied by means of lint and oil-skin, caused a decided improvement to take place. In two months the patient was cured.

*Case* 2.—Specific ulcers of the legs of long standing. This was a case treated in the Edinburgh Infirmary under the care of Dr. Handyside. The man, aged 22, had been suffering for thirteen years from the effects of a kick of a horse, which had produced lacerated wounds on the anterior surface of the tibiæ at their middle third. According to his own account these never entirely healed. On admission, there were several small ill-conditioned sores over the seat of the former injury. From the 9th of May to the 20th June, various metallic washes were applied to the ulcers without success. Then, the ulcers were treated with a strong etherial solution of bromine, which acted as a caustic, and lint steeped in saturated solution of bromine placed over them, covered with oil-skin. Next day the same process was repeated. The application produced pain and intense redness, and after the second time was not repeated. The ulcers healed rapidly afterwards, and cicatrization took place.

*Case* 3.—A case of carbuncle, under the care of Mr. Bennett of Gateshead, which after resisting the hydriodate of potass used externally and internally, was cured by the external use of a solution of bromine—forty minims to the pint of water—in between six and seven weeks. Mr. Bennett has used the lotion of bromine with success in many cases of skin affections, and in cases of purpura.

*Case* 4.—Anomalous syphilitic tubercles of the legs. This was a strong man of 36 years of age, who had been affected with syphilitic and mercurial symptoms for eight years. For six years, he had a discolouration of the skin of the right fore-arm and left thigh, accompanied with pain of the bones at night. For several years, swellings have formed on these parts, which broke, leaving deep ill-conditioned ulcerations. The case was under the care of Mr. Dawson of this town, who commenced the treatment on the 14th of May, 1841. At first, the lotion of bromine—ten minims to the pint, was employed for a month, with the effect of cleaning the ulcers, and removing the discolouration, in a great degree; after this, he had an ointment composed of eight minims of bromine, and half a drachm of the bromide of potassium to an ounce of lard, which was ordered to be well rubbed on daily. Under this applicatiou, without internal treatment, the sores had healed, and the discolouration was removed by the 15th of December. Previous to the commencement of the treatment, the disease had a very formidable appearance.

*Case* 5.—Sarcomatous tumour of the knee, of about the size of an apple, spongy, not painful, in a woman aged 44; removed in a month by frictions of an ointment composed of thirty minims of bromine and a drachm of the bromide of potassium to an ounce of lard.

*Case* 6.—Purulent ophthalmia in a child, where Mr. Brown, of Jarrow, substituted a lotion of bromide of potassium, three grains to the ounce, with success, for the sulphate of zinc previously added.

*Cuse* 7.—Scrofulous ulcer of the leg in a boy, aged 12. Admitted into the Newcastle Infirmary under the care of Sir. J. Fife, July 5th, 1839. A

large foul scrofulous ulcer occupied nearly the whole of the inner aspect of the left leg. There was a similar ulcer, of the size of a half-crown, on the back of the right wrist. There was great pain in the ulcer of the leg, the appetite was bad, and he slept little. The sore on the leg had lasted for three years. Tonics and hydriodate of potass internally were conjoined with the external use of creosote and ioduretted solution of hydriodate of potass. No benefit was produced. In the beginning of December, the bones of the leg and carpus were obviously diseased, and symptoms of hectic appeared. The hydriodate of potass was again tried; no benefit was derived, and the lotion of bromine in the dose of forty minims to the pint of water was applied externally three times a-day by means of lint and oil skin, while the bromide of potassium was given internally.

Under this treatment the ulcers assumed a more healthy aspect, and gradually diminished in size, while their fœtor and pain were lessened. In six weeks the ulcer on the leg was nearly healed; that on the wrist continued open. He was then made an out-patient, and died in the course of the winter, I understand, of diabetes. The bromide of potassium was at first given in the dose of three grains thrice daily, in pill. After three days this dose was increased to four grains, and so every three days, until twenty-four grains were taken daily. Under this treatment the appetite improved, the night-sweats diminished, and the urine increased in quantity. Bromine was several times found in the urine during the treatment.

*Cases* 8, 9, 10, and 11.—These were cases of malignant ulcer of the face, and syphilitic ulcers, where the bromide of mercury was employed internally, in the dose of the eighth of a grain thrice a-day, while the solution of bromine, of the strength described in the preceeding case was used externally. All the patients were men, and the cases were observed in the Newcastle Infirmary. The syphilitic ulcers rapidly healed, but during the internal treatment in three of the cases, symptoms of physiological action were observed exactly like those of corrosive sublimate. Very severe head, chest, and stomach affections were produced. The case of malignant ulcer was somewhat improved, and in this case, although the urine was increased in quantity during a treatment of a month, no head, chest, nor stomach affection was observed. There was slight salivation.

*Case* 13.—Mesenteric tumour in a weaver, aged 22, Edinburgh Infirmary. Here the bromide of iron was used internally without success, so far as the swelling was concerned, but with improvement of the appetite. The dose was twelve grains in twenty pills, two morning and evening, from the 12th to the 21st of August, 1840.

*Cass* 14.—Here the bromide of potassium was employed internally in a case ot scrofulous enlargement of the glands of the neck in a pitman, aged 19, the same salt with bromine being used externally in the form of ointment. Five grains of the salt were given internally every three hours; and the ointment was composed of thirty minims of bromide, and a drachm of the salt to the ounce of lard, and rubbed on thrice a-day. In three weeks the glands were reduced to a third of their size, and some pain which had been felt in the throat was no longer experienced; then the glands began to suppurate; the internal treatment produced no constitutional effect. I owe this case to Mr. Brown of Jarrow.

*Case* 15.—Scrofulous enlargement of the glands of the neck in a girl, aged 16, treated by the bromide of iron internally, and ointment of bromine and hydrobromate of potass externally. The internal remedy was given inthe dose of forty drops of a solution of one drachm in two fluid ounces of water, twice a-day; the external application was of the same strength as in the preceding case. The treatment was continued from the 22d of June, 1841, to the 11th of August, when the general health was much improved, but little effect had been produced on the tumours.

*Case* 16.—Hypertrophy of the submaxillary gland in a youth, aged 18. Treatment and result similar to that in the preceding case. The internal remedy caused some degree of diarrhœa.

*Case* 17.—Case of rupia treated by lotion of bromine externally, and the bromide of iron internally. The patient, aged 23, was treated in the Newcastle Infirmary, by Dr. Cargill. While the sores of one leg were treated by the lotion of bromine of the strength of forty minims to the pint, those of the other leg had nitrate of silver applied; the superiority of the latter application was very evident. The bromine produced soreness, and a serous exudation from the ulcers; the ointment of nitrate of silver, on the other hand, only caused temporary pain, and was soon the means of forming a dry scale. Two drachms of the bromide of iron were dissolved in two fluid ounces of water, and the patient had twenty drops three times a-day. The treatment was commenced on the 22d day of February, 1841. The external treatment by bromine was soon suspended. The dose of the internal remedy was gradually increased to 100 drops three times a-day. This large dose on the 22d of March had produced no unpleasant symptom, but great improvement of the appetite and increase of strength appeared to have resulted from its use. The rupia got well under the application of the nitrate of silver.

*Case* 18.—Chronic rheumatism in a man, aged 24. Here the internal use of the bromide of iron in the dose of six grains thrice a day gave rise to severe pain of the head and chest. (Newcastle Infirmary.)

*Remarks.*—The cases whose prominent features I have thus endeavoured to describe, can be only supposed to give the slightest possible foundation on which to frame inductions with regard to the real therapeutic power of bromine and its compounds. I should be induced to recommend the external use of bromine in scaly dartrous affections of a peculiarly inveterate character, in specific and malignant ulcers where there is defective action, and in the form of lotion the element furnishes an elegant and cleanly application. The solution is slowly changed by the action of light; the hydrobromic acid being formed. Oil-skin should be employed to cover the lint in which the bromine is dipped, in order to prevent the evaporation.

Internally the use of bromine must necessarily be very limited. The sensation which attends the swallowing of it is, I repeat, truly horrid. The bromide of potassium is less powerful than the iodide, and might perhaps be used with advantage, where the latter is apt to disagree with the stomach. The bromide of iron is perhaps the most agreeable of the strong preparations of iron. I have prescribed it frequently as a general tonic, and in hysteria and leucorrhœa. I conceive it to be less liable to decomposition than the iodide. A most exaggerated notion has been entertained of its power. The bromide and sub-bromide of mercury appear to have the good and bad properties of corrosive sublimate and calomel.

*General Conclusions.*

1. Bromine appears to resemble chlorine much more than iodine, in its physiological properties.

2. All the bromides seem to bear a closer relation to the chlorides physiologically than to the iodides.

3. The chemical and physiological relations of the group of halogenous elements and their compounds, are in strict accordance.

4. Although in general the compounds of chlorine, bromine, and iodine with metals appear to resemble other salts of the same bases, in their action on the animal economy, yet we may perceive that the haloid salts are for the most part marked by peculiar resemblances.

5. As far as we can observe, the class of medicinal agents which bromine and its compounds furnish, is intermediate in medicinal action between the two allied groups, but nearer that of chlorine than that of iodine.—DR. GLOVER, *in Edinb. Med. and Surg. Journ.* October 1, 1842.

